# Prevalence, associated factors and management implications of left ventricular outflow tract obstruction in takotsubo cardiomyopathy: a two-year, two-center experience

**DOI:** 10.1186/1471-2261-14-147

**Published:** 2014-10-22

**Authors:** Ole De Backer, Philippe Debonnaire, Sofie Gevaert, Luc Missault, Peter Gheeraert, Luc Muyldermans

**Affiliations:** Department of Cardiology, Ghent University Hospital, De Pintelaan 185, B-9000 Ghent, Belgium; Department of Cardiology, AZ Sint-Jan Hospital, Bruges, Belgium

**Keywords:** Takotsubo cardiomyopathy, Apical ballooning, Outflow tract obstruction, Systolic anterior motion, Cardiogenic shock

## Abstract

**Background:**

Some patients with Takotsubo cardiomyopathy (TTC) develop cardiogenic shock due to left ventricular outflow tract (LVOT) obstruction – there is, however, a paucity of data regarding this condition.

**Methods:**

Prevalence, associated factors and management implications of LVOT obstruction in TTC was explored, based on two-year data from two Belgian heart centres.

**Results:**

A total of 32 patients with TTC were identified out of 3,272 patients presenting with troponin-positive acute coronary syndrome. In six patients diagnosed with TTC (19%), a significant LVOT obstruction was detected by transthoracic echocardiography. Patients with LVOT obstruction were older and had more often septal bulging, and presented more frequently in cardiogenic shock as compared to those without LVOT obstruction (P < 0.05). Moreover, all patients with LVOT obstruction showed systolic anterior motion (SAM) of the anterior mitral valve leaflet, which was associated with a higher grade of mitral regurgitation (2.2±0.7 vs. 1.0±0.6, P<0.001). Adequate therapeutic management including fluid resuscitation, cessation of inotropic therapy, intravenous β-blocker, and the use of intra-aortic balloon pump resulted in non-inferior survival in TTC patients with LVOT obstruction as compared to those without LVOT obstruction.

**Conclusions:**

TTC is complicated by LVOT obstruction in approximately 20% of cases. Older age, septal bulging, SAM-induced mitral regurgitation and hemodynamic instability are associated with this condition. Timely and accurate diagnosis of LVOT obstruction by echocardiography is key to successful management of these TTC patients with LVOT obstruction and results in a non-inferior outcome as compared to those patients without LVOT obstruction.

**Electronic supplementary material:**

The online version of this article (doi:10.1186/1471-2261-14-147) contains supplementary material, which is available to authorized users.

## Background

Takotsubo cardiomyopathy (TTC) – also called apical ballooning syndrome – is an increasingly reported clinical entity characterized by transient severe systolic heart failure that mimics an acute myocardial infarction in the absence of obstructive coronary artery disease [1, 2]. This condition predominantly affects postmenopausal women and emotional/physical stress at onset is common, although a triggering event is not always present. Postulated pathogenic mechanisms include multivessel epicardial vasospasm, coronary microvascular dysfunction, and catecholamine-triggered myocyte injury. Other studies hypothesize that in the presence of increased catecholamine levels, a dynamic intraventricular pressure gradient develops, resulting in subendocardial ‘stunning’ of the left ventricular (LV) apical region [1–6].

Importantly, some patients with TTC develop cardiogenic shock due to severe systolic dysfunction or left ventricular outflow tract (LVOT) obstruction. There is, however, a paucity of data regarding this latter condition. Accordingly, we explored the prevalence and characteristics of TTC in a population presenting with troponin-positive acute coronary syndrome (ACS) – with focus on LVOT obstruction and its management.

## Methods

### Study population

The study population consisted of all patients referred with troponin-positive ACS to two high-volume catheterisation laboratories (Ghent University Hospital, AZ Sint-Jan Bruges) in Belgium in a period of 24 consecutive months. The diagnosis of TTC was based on the Mayo-criteria [5]: (1) transient hypokinesia, akinesia, or dyskinesia of the LV mid segments with or without apical involvement – regional wall motion abnormalities extend beyond a single epicardial distribution; (2) absence of obstructive coronary artery disease or angiographic evidence of acute plaque rupture; (3) new electrocardiographic abnormalities (either ST-segment elevation and/or T-wave inversion) or elevated cardiac troponin; (4) absence of a pheochromocytoma, myocarditis or hypertrophic cardiomyopathy.

Clinical, biochemical, electrocardiographic, echocardiographic and angiographic data were retrospectively collected. Clinical data included demographics, cardiovascular risk factors, presenting symptoms and emotional or physical triggers. The peak Troponin-T (TnT)-value was measured as a marker of myocyte necrosis. Coronary and LV angiography were performed within 72 hours after onset of symptoms. LV angiograms were used to calculate LV ejection fraction (LVEF) and detect regional wall motion abnormalities. Based on review of the echocardiographic images, TTC patients were further dichotomized depending on the presence of significant LVOT obstruction. Due to the retrospective nature of this study, the choice of treatment was at the discretion of the treating physician. Approval for this study was provided by the local Ethical Committee of Ghent University Hospital (Ghent, Belgium) and all patients gave written informed consent for the use of anonymous clinical, procedural, and follow-up data.

### Echocardiography

Within 24 hours of admission, all patients with suspected TTC underwent transthoracic echocardiography. A follow-up echocardiographic study was performed in most patients at random intervals, most often at day 4–14 (at discharge), at 4–8 weeks, and approximately one year after the acute phase. Standard gray-scale and color-Doppler images were acquired with ECG-triggering and in cine-loop format for on-line analysis. In particular, septal wall thickness was measured at parasternal long axis view and septal bulging was defined as basal interventricular septum (IVS) thickness ≥12 mm. The same view was used for detection of systolic anterior motion (SAM) of the anterior mitral valve leaflet. LVEF was evaluated using Biplane Simpson method, as recommended [7]. In addition, regional wall motion abnormalities were assessed. Color Doppler was used to identify turbulent flow, suggesting increased pressure gradient in the LV. To further identify significant peak pressure gradients (defined as >20 mmHg), pulsed wave Doppler was used, set above the mitral leaflets tips (assessment of intraventricular pressure gradient) and into the LVOT (assessment of LVOT pressure gradient/obstruction). The final gradient was calculated using the modified Bernoulli equation based on maximal flow velocities derived from continuous wave Doppler imaging. As previously reported, mitral regurgitation (MR) severity (grade 0–4) was assessed using an integrative approach based on vena contracta width, color Doppler jet area, or by quantitative approach whenever possible (Doppler-volumetric/proximal isovelocity surface area) [8].

### Statistical analysis

Continuous variables are reported as means ± standard deviation, unless otherwise specified. Categorical data are reported as absolute values and percentages. Continuous and categorical variables were compared by (un)paired *t* test, χ^2^ test and Fisher test, as appropriate. All data were analysed using SPSS version 20.0 (SPSS Inc., Chicago, IL, USA). *P* values <0.05 were considered statistically significant.

## Results

### Overall study population

**Figure 1:**
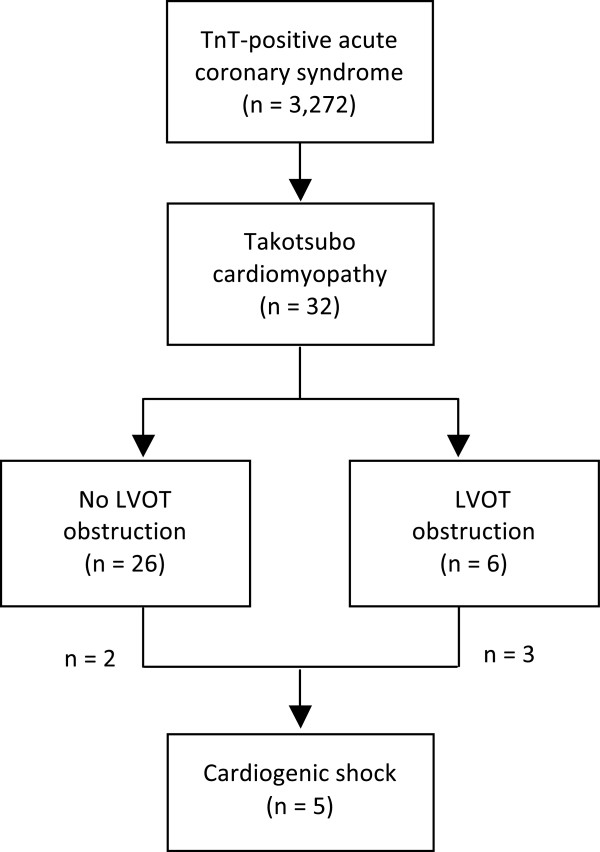
**Prevalence of takotsubo cardiomyopathy in a population presenting with troponin-positive acute coronary syndrome.**

Out of 3,272 patients with troponin-positive ACS referred for coronary angiography, a total of 32 patients were identified with TTC – indicating an overall prevalence of 1.0% (Figure 1).

As shown in Table 1, TTC patients were predominantly older women (66 ± 15 years, 94% female). The overall cardiovascular risk profile was rather low, in line with the absence of significant coronary artery lesions on coronary angiography. The most common presenting symptoms were chest pain (n = 17; 53%), respiratory distress (n = 8; 25%), and cardiogenic shock (n = 5; 16%). Two other patients presented with ventricular tachycardia (VT) and fibrillation (VF), both were successfully resuscitated. In 22 patients, a stressful event preceding the acute phase of TTC could be identified. These events were considered emotionally mediated in 9 patients (28%) or alternatively due to a physical trigger in 13 other patients (41%) – these circumstances are detailed in Additional file 1: Table S1. On admission, ST-segment elevation mimicking acute anterior myocardial infarction was present in almost half of the patients (n = 15, 47%), while 16 patients (50%) presented with other ST/T abnormalities. On admission, TnT was elevated in 28 patients (88%) and peak in-hospital TnT was 0.93 ± 0.90 ng/mL (range 0.1 to 3.6 ng/mL). Echocardiography and LV angiography revealed a typical pattern of ‘apical ballooning’ with akinesia of the mid/apical LV segments and compensatory hyperkinesia of the basal segments in the majority of patients (n = 30). Only two patients presented with ‘inverse’ TTC or mid-ventricular ballooning, ie. akinesia of the mid LV segments. Per patient clinical, biochemical, electro-/echo-cardiographic and angiographic data are available in Additional file 1: Table S1.

**Table 1:** **Baseline characteristics of patients with takotsubo cardiomyopathy**

	Total (n = 32)	No LVOT obstruction (n = 26)	LVOT obstruction (n = 6)	***P***-value
Age (years), mean ± SD	66 ± 15	64 ± 15	77 ± 7	0.047*
Female, *n* (%)	30 (93.8)	24 (92.3)	6 (100)	1.000
Risk factors, *n* (%)				
Hypertension	18 (56.2)	13 (50.0)	5 (83.3)	0.196
Hypercholesterolemia	8 (25.0)	5 (19.2)	3 (50.0)	0.296
Diabetes mellitus	3 (9.4)	2 (7.7)	1 (16.7)	1.000
Trigger, *n* (%)				
Physical stress	13 (59.1)	12 (46.2)	1 (16.7)	0.387
Emotional stress	9 (40.9)	8 (30.8)	1 (16.7)	0.850
Presenting symptom, *n* (%)				
Chest pain	17 (53.1)	15 (57.7)	2 (33.3)	0.383
Respiratory distress	8 (25.0)	7 (26.9)	1 (16.7)	1.000
Cardiogenic shock	5 (15.6)	2 (7.7)	3 (50.0)	0.034*
VT/VF	2 (6.2)	2 (7.7)	0 (0.0)	1.000
ECG, *n* (%) *or* mean ± SD				
ST-elevation	17 (53.1)	13 (50.0)	4 (66.7)	0.659
ST-depression/negT	14 (43.8)	12 (46.2)	2 (33.3)	0.672
QRS (ms)	97 ± 10	98 ± 10	92 ± 7	0.179
QTc (ms)	421 ± 30	418 ± 27	435 ± 43	0.223
TnT (ng/mL), mean ± SD	0.93 ± 0.90	0.99 ± 0.96	0.68 ± 0.50	0.462
TTE, *n* (%) *or* mean ± SD				
LVEF (%)^#^	40.5 ± 10.3	41.1 ± 11.0	38.0 ± 5.8	0.512
IVS (mm)	10.8 ± 1.7	10.5 ± 1.4	12.0 ± 2.1	0.044*
Septal bulge	12 (37.5)	7 (26.9)	5 (83.3)	0.018*
SAM	6 (18.8)	0 (0.0)	6 (100)	< 0.001*
MR grade	1.25 ± 0.73	1.0 ± 0.6	2.2 ± 0.7	< 0.001*
Therapeutic options, *n* (%)				
Inotropics i.v.	9 (28.1)	7 (26.9)	2 (33.3)	1.000
Beta-blocker i.v.	2 (6.2)	0 (0.0)	2 (33.3)	0.030*
IABP	9 (28.1)	7 (26.9)	2 (33.3)	1.000
Recuperation, mean ± SD				
LVEF ≥55% (days)	19 ± 12	18 ± 11	23 ± 16	0.479

Continuous variables are reported as means ± SD.; categorical variables are reported as absolute values and percentages. Continuous and categorical variables were compared by use of (un)paired t test, χ^2^ test and Fisher test, as appropriate. *Abbreviations*: *VT/VF* ventricular tachycardia/fibrillation, *TnT* troponin T, *TTE* transthoracic echocardiography, *LVEF* left ventricular ejection fraction, *IVS* interventricular septum thickness, *SAM*, systolic anterior motion, *MR* , mitral regurgitation, *IABP* intraaortic balloon pump. ^#^LVEF as calculated on LV angiogram (and confirmed on transthoracic echocardiography).

*P-value < 0.05.

### Patients without vs. with LVOT obstruction

As shown in Table 1, a total of six patients (19%) were identified with significant LVOT obstruction and no patients were found to have a significant intraventricular pressure gradient. The TTC patient population was dichotomized based on the absence or presence of LVOT obstruction. The latter group was significantly older and presented more often with hemodynamic instability as compared to patients without LVOT obstruction (n = 26, 81%). In addition, a higher basal IVS thickness leading to increased prevalence of septal bulging was found. Patients with LVOT obstruction all showed SAM and consequently more severe MR as compared to patients without LVOT obstruction (Table 1). Of note, none of these patients had a familial history of hypertrophic cardiomyopathy. Both patient groups without vs. with LVOT obstruction had similar peak TnT and mean LVEF at admission. No LVOT obstruction was found in the two patients that were resuscitated because of VT/VF.

### LVOT obstruction and therapeutic management

Similar proportions of TTC patients without vs. with LVOT obstruction were initially treated with i.v. inotropics (27% vs. 33%, respectively; Table 1). However, inotropic therapy was immediately stopped in the two patients with LVOT obstruction from the moment of diagnosis by echocardiography. Patients without LVOT obstruction were given inotropic agents for a maximum duration of five days. In addition, an intra-aortic balloon pump (IABP) was used in 9 TTC patients (28%) because of severe systolic dysfunction (LVEF < 35%, n = 4), LVOT obstruction (n = 2), or non-specified reason(s) (n = 3). Only two patients received β-blocker i.v. – both patients were diagnosed with severe LVOT obstruction (pressure gradient ≥40 mmHg). Per patient therapeutical management data are available in Additional file 1: Table S1.

### Patient outcome

A total of two TTC patients (32, 6%) without LVOT obstruction died in the acute setting due to refractory cardiogenic shock (n = 1) or acute respiratory distress syndrome (n = 1, see Additional file 1: Table S1). No mortality occurred in the group of patients diagnosed with LVOT obstruction. There was no residual dynamic LVOT pressure gradient detected at discharge in any of the patients initially presenting with LVOT obstruction. In accordance, there was no more SAM of the anterior mitral valve leaflet, resulting in an important reduction of MR severity (mean MR grade 1.2 ± 0.6 *vs.* 2.2 ± 0.7). Recovery of LV systolic function – defined as LVEF ≥55% – was observed within 19 ± 12 days (range 5 to 42 days – based on data obtained in 24 patients, see Additional file 1: Table S1). At medium to long-term follow-up, two patients experienced recurrence of TTC at 0.9 and 5.2 years after the first episode, while on oral β-blocker therapy. No evident relation was found between baseline clinical features, ECG pattern, TnT levels, and/or outcome (data not shown).

## Discussion

The main findings of this study are: (1) LVOT obstruction is not uncommon in TTC, (2) patients with LVOT obstruction have a different clinical and echocardiographic baseline profile, and (3) an adequate and tailored therapy in TTC patients with LVOT obstruction results in a non-inferior outcome as compared to those without LVOT obstruction.

In line with previous findings [3–5, 9], we report an overall prevalence of TTC of 1.0% in patients presenting with troponin-positive ACS – this prevalence is even higher when considering only female patients, as TTC predominantly occurs in postmenopausal women. TTC is not necessarily a benign disease, considering the occurence of cardiogenic shock, life-threatening arrhythmias, and sudden cardiac death [10–12] as well as persistent wall motion abnormalities with delayed recovery [13, 14]. Interestingly, we noted a co-incidence of LVOT obstruction in approximately one out of five subjects (19%) presenting with TTC – this is in line with other studies reporting a 5-25% prevalence of LVOT obstruction in TTC patients [6, 15–18].

In addition, our study confirms the presence of a characteristic baseline profile in TTC patients with LVOT obstruction – as previously reported by El Mahmoud et al. [18] – and adds data regarding trigger, presenting symptoms and therapeutic options. In particular, patients with LVOT obstruction were older and nearly all presented a septal bulge, associated with SAM of the mitral valve and significant MR. This morphological pattern of the IVS is mostly present in elderly patients with a medical history of hypertension and seems to be a predisposing and/or contributing factor to the development of LVOT obstruction in TTC patients, thereby mimicking a pattern of hypertrophic obstructive cardiomyopathy.

In 2001, Villareal et al. reported for the first time LVOT obstruction in three patients with TTC [19]. Other groups could confirm these pathologic findings, especially in women with a mid-ventricular septal thickening, suggesting that this could be an important factor in the development of this syndrome [20]. It was hypothesized that in the presence of increased concentrations of catecholamines – caused by a stressful event – this mid-ventricular septal thickening could lead to the development of a severe transient LV mid-cavity obstruction, resulting in ‘apical stunning’ (unrelated to a specific coronary artery territory). Still, it remains unclear whether the observed LVOT pressure gradient is a ‘consequence’ rather than a ‘cause‘ of TTC, also given the fact that LVOT obstruction does not occur in all TTC patients [21]. Remarkably, approximately half of our TTC patients with LVOT obstruction exhibited cardiogenic shock at admission, suggesting that this mechanism plays a detrimental role in the further clinical evolution of this disease. Of note, none of the TTC patients with LVOT obstruction died in our cohort and all patients had full LVEF recovery, which was accompanied by disappearance of the dynamic LVOT pressure gradient, disappearance of SAM and MR reduction.

### Clinical implications

**Figure 2:**
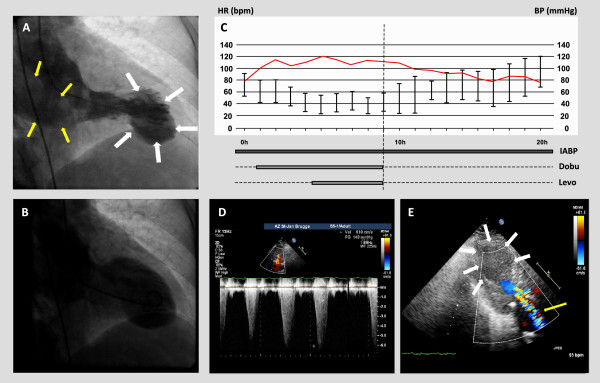
**Diagnostic evaluation and treatment of a patient with takotsubo cardiomyopathy and severe left ventricular outflow tract (LVOT) obstruction.** A 74-year old woman was admitted with ST-segment elevation in the precordial leads and in cardiogenic shock. Panel **A-B**: Left ventricular angiography shows a typical pattern of ‘apical ballooning’ at systole **(panel A)** when compared to diastole **(panel B)**. Panel **C**: Intra-aortic balloon pump counter-pulsation therapy was initiated in the cath-lab. Because of refractory shock, dobutamine (dobu) and norepinephrine (levo) were started at the ICU. Cessation of inotropic therapy after echocardiographic diagnosis of LVOT obstruction resulted in recovery of blood pressure. Panel **D**: Severe LVOT obstruction was identified on continuous wave Doppler echocardiography (end-systolic pressure gradient 149 mmHg). Panel **E**: Echocardiography confirming the presence of apical akinesia or ‘apical ballooning’. HR: heart rate (beats per minute); BP: blood pressure (mmHg). The white arrows indicate ‘apical ballooning’; the yellow arrows indicate systolic anterior motion (SAM)-induced mitral regurgitation.

**Figure 3:**
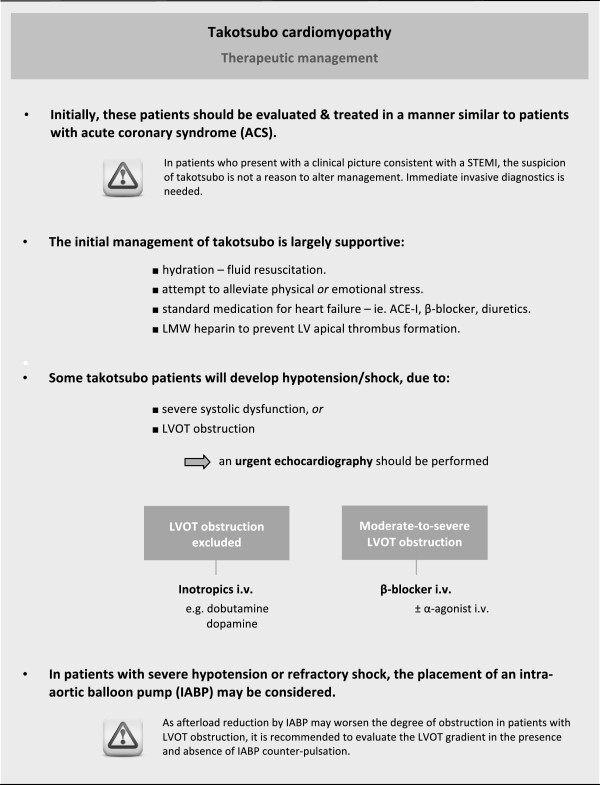
**Therapeutic management of takotsubo cardiomyopathy – a practical flow chart.**

Identification of TTC patients at risk for LVOT obstruction is of critical importance as its presence has important therapeutical management implications. In particular, older age and presence of septal bulge are associated with LVOT obstruction and may be considered potential risk factors. In addition, hemodynamic instability at presentation should raise suspicion of LVOT obstruction in these patients. In fact, the use of inotropic agents should be avoided in patients with LVOT obstruction, particularly when hemodynamic instability is present, as inotropic therapy may increase LVOT obstruction and worsen cardiogenic shock [22–25]. Administration of β-blockers has been shown not to be detrimental and potentially of benefit to alleviate LVOT obstruction in these patients [26]. However, patients without significant LVOT obstruction who are hypotensive due to severe LV dysfunction can be treated with inotropes such as dobutamine and dopamine, and in these cases, use of β-blockers is contraindicated [22]. Therefore, we suggest that transthoracic echocardiography should be systematically performed at admission in all patients presenting with TTC in order to identify presence of LVOT obstruction, especially when additional risk factors are present. The importance of echocardiography for the successful management of TTC patients in cardiogenic shock is illustrated by patient case #12, in which the detection of severe LVOT obstruction – evidenced by a peak end-systolic pressure gradient of 149 mmHg – led to cessation of all inotropic support and, ultimately, the survival of this patient who was admitted with profound cardiogenic shock (Figure 2).Based on our findings and previous reports, we propose a practical flow chart for the management of patients with TTC in Figure 3. In general, the initial management of TTC should be largely supportive, including adequate hydration in non-acute heart failure patients and an attempt to alleviate the triggering physical or emotional stress. When confronted with TTC patients with hypotension/shock, urgent echocardiographic evaluation is indispensable in order to evaluate LV systolic function as well as the potential presence of LVOT obstruction. In patients with hypotension and moderate-to-severe LVOT obstruction, inotropic agents and vasodilators should be avoided and use of β-blockers warrants consideration. In patients with severe hypotension or refractory shock, the use of IABP may be considered. As there is a potential risk that afterload reduction from the IABP may worsen the degree of obstruction in patients with LVOT obstruction, we recommend evaluating the LVOT gradient in the presence and absence of IABP counter-pulsation

Controlled data defining the optimal medical regimen for TTC patients after the acute setting are lacking. However, it is reasonable to treat these patients with standard medications for LV systolic dysfunction until LV systolic function fully recovers, including angiotensin converting enzyme inhibitors, β-blockers and diuretics as appropriate. Moreover, a short course of low molecular weight heparins to prevent LV apical thrombus formation may be recommended [22]. Data that would support the persistent use of oral β-blocker therapy in order to prevent long-term re-occurence of TTC are currently not available.

### Study limitations

We recognize this study is hampered by its retrospective and observational nature. In addition, assessment of independent predictors of LVOT obstruction is not possible, given the limited number of cases presenting with LVOT obstruction. However, our results are hypothesis generating and the impact of LVOT obstruction on therapeutic management and outcome of patients presenting with TTC needs further prospective study.

## Conclusions

LVOT obstruction complicating TTC is a common transient phenomenon, suspected if presence of hemodynamic instability. Older age and presence of a septal bulge are predisposing factors for LVOT obstruction development. Timely and accurate diagnosis by echocardiography is key to successful and tailored management of TTC with LVOT obstruction and results in non-inferior outcome as compared to TTC patients without this condition.

## Electronic supplementary material

Additional file 1: **Detailed characteristics of all patients presenting with Takotsubo cardiomyopathy (n = 32).** (PDF 107 KB)

## Abbreviations

TTC: Takotsubo cardiomyopathy
LVOT: Left ventricular outflow tract
SAM: Systolic anterior motion
LV: Left ventricular
ACS: Acute coronary syndrome
TnT: Troponin-T
LVEF: Left ventricular ejection fraction
IVS: Interventricular septum
MR: Mitral regurgitation
VT: Ventricular tachycardia
VF: Ventricular fibrillation
IABP: Intra-aortic balloon pump.
