# Supplementary material for: Prevalence, associated factors and management implications of left ventricular outflow tract obstruction in takotsubo cardiomyopathy: a two-year, two-center experience
Source: BMC Cardiovasc Disord. 2014 Oct 22;14:147. doi: 10.1186/1471-2261-14-147 (PMC4210484; doi:10.1186/1471-2261-14-147)
Supplement: Supplementary file 1 — Additional file 1: Detailed characteristics of all patients presenting with Takotsubo cardiomyopathy (n = 32). (PDF 107 KB) [file 12872_2014_791_MOESM1_ESM.pdf]

Supplementary Table 1A

		Comorbidities					ECG				Echocardiography					Therapy			
Patient #	Age/Sex	HT	HC	DM	Trigger	Presenting symptoms	QRS ms	ST/T	QTc ms	Max TnT ng/mL	LVEF [#] %	IVS mm	$LVOT_{grad}$ mmHg	SAM	MR	Inotrop	BBL	IABP days	Time to LVEF recup days
1	65/F	+	+	-	Asthma exacerbation	Resp distress	94	Neg T	393	0.2	48	13	< 20	-	0	-	-	-	14d
2	58/F	-	-	-	-	Card shock	104	Neg T	441	2.1	22	10	< 20	-	1+	+	-	2d	†
3	38/F	-	-	+	Anaphylaxis – Epipen®	VF	99	STE	464	0.3	25	9	< 20	-	2	-	-	-	10d
4	56/F	+	-	-	Conflict at work	Chest pain	82	Neg T	422	1.3	42	12	< 20	-	1	-	-	-	28d
5	68/F	+	-	-	Tragic news – brother †	Chest pain	111	Neg T	430	0.1	53	11	< 20	-	0	-	-	-	-
6	57/M	+	+	-	-	Resp distress	84	STE	401	3.5	40	13	< 20	-	1	-	-	-	28d
7	78/F	-	+	-	-	Chest pain	87	STE	492	0.4	31	8	28	+	2	-	-	-	-
8	66/F	+	-	-	Missed appointment	Chest pain	93	STE	400	1.0	34	12	40	+	3	-	+	-	42d
9	77/F	-	+	-	Dental surgery	Chest pain	117	NegT	414	0.6	46	10	< 20	-	1	-	-	-	42d
10	60/F	-	-	-	-	Resp distress	98	QS_{V1-4}	400	2.3	30	9	< 20	-	0	+	-	-	†
11	52/F	+	-	-	RF ablation (PVI)	Resp distress	124	LBBB	386	0.3	48	12	< 20	-	1	-	-	-	21d
12	74/F	+	-	-	-	Card shock	91	STE	417	1.3	37	13	149	+	2	+	-	4d	28d
13	51/F	+	-	-	Exercise - tachycardia	Resp distress	95	-	393	0.9	48	11	< 20	-	1	-	-	-	-
14	58/F	-	-	-	Dehydration	Chest pain	104	STE	405	0.5	34	10	< 20	-	2	-	-	-	21d
15	67/F	-	-	-	Abdominal surgery	Chest pain	89	STE	382	0.5	45	11	< 20	-	1	-	-	-	14d
16	31/F	-	-	-	-	Card shock	95	NegT	460	1.6	19	9	< 20	-	1+	+	-	5d	14d

Age: years. Sex: F, female; M, male. Comorbidities: HT, hypertension; HC, hypercholesterolemia; DM, diabetes mellitus. Trigger: RF, radiofrequency; PVI, pulmonary vein isolation. Presenting symptoms: Resp distress, respiratory distress; Card shock, cardiogenic shock; VT/VF, ventricular tachycardia/fibrillation. ECG: Neg T, negative T-waves; STE, ST elevations; LBBB, left bundle branch block; STD, ST depressions. Max TnT, maximal troponin T value (ng/ml; cut-off value 0.035). Echocardiography: LVEF, LV ejection fraction (%); IVS, interventricular septum (mm; septal bulge if ≥ 12 mm). LVOTgrad, left ventricular outflow tract gradient (mmHg); SAM, systolic anterior motion of the anterior mitral valve leaflet; MR, mitral regurgitation grade on a scale of 4. Therapy: Inotrop, inotropics i.v.; BBL, beta-bocker i.v.; IABP, intra-aortic balloon pump. Time to LVEF recup, LVEF recuperation = LVEF ≥ 55% (days). [#] LVEF as calculated on LV angiogram (and confirmed on transthoracic echocardiography).

Supplementary Table 1B

Patient #	Age/Sex	Comorbidities HT	HC	DM	Trigger	Presenting symptoms	ECG QRS ms	ST/T	QTc ms	Max TnT ng/mL	Echocardiography LVEF[#] %	IVS mm	$LVOT_{grad}$ mmHg	SAM	MR	Therapy Inotrop	BBL	IABP days	Time to LVEF recup days
17	36/M	+	-	-	-	Chest pain	91	Neg T	448	3.6	40	9	< 20	-	1	-	-	-	10d
18	76/F	-	+	-	Conflict with husband	Chest pain	96	STE	399	0.5	38	10	< 20	-	1	+	-	3d	5d
19	74/F	+	-	-	Cardioversion – anxiety	Chest pain	113	STE	403	0.9	61	13	< 20	-	1	-	-	-	-
20	93/F	+	-	-	Chronic stress	Chest pain	100	STE	417	1.0	43	9	< 20	-	2	-	-	-	28d
21	83/F	+	-	+	-	Resp distress	97	Neg T	392	1.2	48	12	32	+	2	-	-	-	-
22	84/F	+	+	-	Acute low back pain	Card shock	83	Neg T	425	0.3	38	14	35	+	2+	+	-	-	7d
23	83/F	-	-	-	Car accident	Chest pain	88	STD	427	0.7	34	10	< 20	-	1	+	-	3d	5d
24	71/F	-	-	-	Acute diverticulitis	Chest pain	98	STE	430	1.5	35	9	< 20	-	1	-	-	-	14d
25	52/F	-	-	-	Panic attack – house fire	Chest pain	95	STD	412	0.2	44	8	< 20	-	1+	-	-	-	5d
26	58/F	+	-	-	Thyroid surgery	Chest pain	92	Neg T	397	0.1	63	12	< 20	-	1	-	-	-	7d
27	75/F	+	+	-	-	Card shock	101	STE	484	0.8	40	13	64	+	2	-	+	3d	14d
28	71/F	-	-	-	Myasthenia gravis crisis	Resp distress	96	STE	470	0.5	39	10	< 20	-	0	+	-	4d	28d
29	83/F	-	-	+	Fall at home – anxiety	Chest pain	89	STE	402	1.3	28	11	< 20	-	1	+	-	3d	42d
30	69/F	+	-	-	-	VT	105	Neg T	474	0.7	55	12	< 20	-	1+	-	-	-	10d
31	74/F	+	-	-	COPD exacerbation	Resp distress	94	STE	405	0.3	39	9	< 20	-	1	-	-	3d	-
32	76/F	+	+	-	Migraine attack	Chest pain	92	STD	394	1.1	50	11	< 20	-	1	-	-	-	14d

Age: years. Sex: F, female; M, male. Comorbidities: HT, hypertension; HC, hypercholesterolemia; DM, diabetes mellitus. Trigger: COPD, chronic obstructive pulmonary disease. Presenting symptoms: Resp distress, respiratory distress; Card shock, cardiogenic shock; VT/VF, ventricular tachycardia/fibrillation. ECG: Neg T, negative T-waves; STE, ST elevations; LBBB, left bundle branch block; STD, ST depressions. Max TnT, maximal troponin T value (ng/ml; cut-off value 0.035). Echocardiography: LVEF, LV ejection fraction (%); IVS, interventricular septum (mm; septal bulge if ≥ 12 mm). LVOTgrad, left ventricular outflow tract gradient (mmHg); SAM, systolic anterior motion of the anterior mitral valve leaflet; MR, mitral regurgitation grade on a scale of 4. Therapy: Inotrop, inotropics i.v.; BBL, beta-bocker i.v.; IABP, intra-aortic balloon pump. Time to LVEF recup, LVEF recuperation = LVEF ≥ 55% (days). [#] LVEF as calculated on LV angiogram (and confirmed on transthoracic echocardiography).
